# A Novel Mesoscale Eddy Identification Method Using Enhanced Interpolation and A Posteriori Guidance

**DOI:** 10.3390/s25020457

**Published:** 2025-01-14

**Authors:** Lei Zhang, Xiaodong Ma, Weishuai Xu, Xiang Wan, Qiyun Chen

**Affiliations:** 1 Department of Military and Marine Mapping, Dalian Naval Academy, Dalian 116021, China; 2 Dalian Naval Academy Cadet Brigade, Dalian 116000, China; 17640339665@163.com (X.M.); xuweishuai2022@163.com (W.X.); wan759189627@163.com (X.W.); 18940890078@163.com (Q.C.)

**Keywords:** mesoscale eddy, identification method, deep learning, YOLO

## Abstract

Mesoscale eddies are pivotal oceanographic phenomena affecting marine environments. Accurate and stable identification of these eddies is essential for advancing research on their dynamics and effects. Current methods primarily focus on identifying Cyclonic and Anticyclonic eddies (CE, AE), with anomalous eddy identification often requiring secondary analyses of sea surface height anomalies and eddy center properties, leading to segmented data interpretations. This study introduces a deep learning model integrating multi-source fusion data with a Squeeze-and-Excitation (SE) attention mechanism to enhance the identification accuracy for both normal and anomalous eddies. Comparative ablation experiments validate the model’s effectiveness, demonstrating its potential for more nuanced, multi-source, and multi-class mesoscale eddy identification. This approach offers a promising framework for advancing mesoscale eddy identification through deep learning.

## 1. Introduction

Mesoscale eddies represent a significant area of study within marine science, exhibiting a widespread distribution across the world’s oceans. The life cycle of these eddies typically extends from several tens to hundreds of days, with their spatial effect ranging from tens to hundreds of kilometers [1]. Their formation and evolution are significantly impacted by the Coriolis force resulting from Earth’s rotation, which imparts different rotational directions and dynamic characteristics [2]. In the Northern Hemisphere, mesoscale eddies predominantly manifest as cyclonic eddies (cold eddies), characterized by counterclockwise rotation and relatively low core water temperatures. Conversely, anticyclonic eddies, which rotate clockwise (i.e., warm eddies), exhibit a higher central water temperature [3].

With the advent of ocean observation satellites in the 1990s, ocean mesoscale eddies became more accessible to scientific inquiry, facilitating a series of systematic studies and discussions regarding these phenomena [4,5,6]. Among the various theoretical investigations, the identification of mesoscale eddies is a critical prerequisite for subsequent analyses following the acquisition of comprehensive satellite imagery, serving as the foundation for the rigor of later research endeavors. Consequently, numerous scholars have explored this topic from diverse perspectives, including physical constraints, graphical processing, deep learning, and fluid mechanics. Okubo et al. [7] and Weiss et al. [8] proposed a mesoscale eddy identification algorithm known as the Okubo-Weiss (OW) method, which is grounded in the statistical parameters of eddy physics. Additionally, an enhanced Q-criterion method was developed by Hunt et al. [9], Liu et al. [10], Chong et al. [11], and Jeong et al. [12]. Physical parameter methods, such as the ω-criterion, δ-criterion, and λ-criterion methods, rely on the inherent physical properties of the flow field, establishing thresholds based on the state (deformation or rotation characteristics) at a specific moment to extract reliable eddy information through constraint screening techniques. Although these physical parameter methods are advantageous due to their low computational cost, the emergence of alternative identification algorithms has increasingly underscored issues related to high misjudgment rates and challenges in accurately determining threshold values. As a result, the downflow field geometry method, advocated by McWilliams et al. [13], has gained prominence among researchers focused on mesoscale vorticity. This method, initially applied in the realm of topological graphics, has rapidly evolved due to its independence from the specification of characteristic parameters and its minimal data requirements concerning the flow field. Notably, the Winde-Angle (WA) algorithm introduced by Sadarjoen et al. [14] and the refined Vector Geometry (VG) algorithm introduced by Nencioli et al. [15] and Dong et al. [16] emerged as leading approaches, targeting the geometric contour features of mesoscale eddies. A continuous and symmetrical velocity vector field surrounding a velocity extremum is regarded as the undetermined target, with the final profile and eddy center established by constraining the velocity and expanding the range. This foundational algorithm has been further optimized and applied by Chelton et al. [1] and Faghmous et al. [17], thereby affirming its stable position within the domain of mesoscale eddy identification.

The emergence of new technologies has consistently necessitated the updating and iteration of traditional algorithms. In recent years, deep learning algorithms have demonstrated significant efficacy in the analysis and application of big data, which is particularly appealing in the marine domain, where the integration of multi-source data and extensive nonlinear feature analysis is required. Consequently, a plethora of reliable depth identification algorithms have been developed. Lguensat et al. [18] introduced Eddy Net, which utilizes deep learning-based image segmentation technology to analyze Sea Level Height datasets (SSH). Duo et al. [19] proposed a mesoscale eddy identification and positioning algorithm (i.e., OEDNet) grounded in object detection networks. Xu et al. [20] developed the PSPNet network, employing the principles of semantic segmentation, and achieved commendable performance in high-accuracy applications for scroll identification and detection. Bai et al. [21] proposed the StreamPath Region-based Convolutional Neural Network (SP-RCNN), an identification method that enhances the deep network’s capacity to express eddy features and improve identification accuracy by transforming flow field data into a novel perspective of flow paths. Du et al. [22] introduced an identification algorithm named DeepEddy, which is based on Synthetic Aperture Radar (SAR) image data. This method employs a unique hierarchical feature learning model to extract invariant and high-level eddy features, ultimately yielding medium-scale eddy identification results with high accuracy through the nonlinear transformation of a binary hash layer. Xie et al. [23] proposed a dual U-net network that is used to identify the warm and cold vortices in the South China Sea and discuss further the statistical characteristics of the anomalous vortices.

Current identification and tracking algorithms primarily depend on predetermined settings, specific thresholds, and quantified parameters (e.g., eddy radius, center height, life cycle) to filter potential eddies. These parameters, often hard to validate and based on general sea surface information, may lead to inaccurate results since navigation measurements such as Argo do not cover all eddies. To solve this, an integrated method guided by posterior results is needed to judge and eliminate errors beforehand. Given the complexity and lack of established validity among mesoscale eddy parameters, this study utilizes deep learning’s “black box” to autonomously learn and distinguish valid parameters, which can be efficiently integrated into existing eddy identification algorithms.

**Figure 1:**
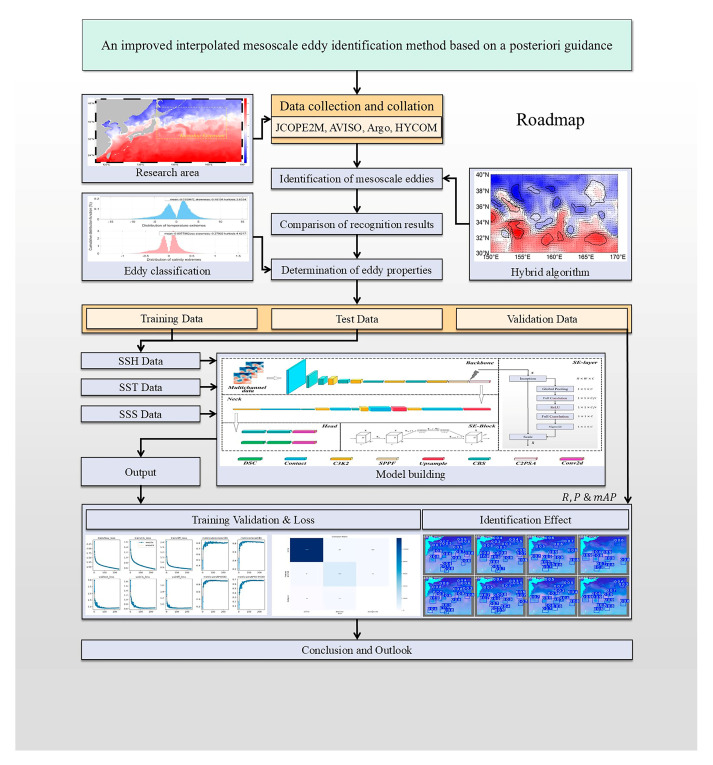
Schematic of the methodological framework.

In summary, the research concepts presented in this study can be categorized into several modules: (1) large-area coarse screening of mesoscale eddies; (2) development of a method for assessing the authenticity of eddies; (3) utilizing the results from modules (1) and (2) to establish a dataset of eddies characterized by unified properties of both sea surface and underwater environments, including normal and abnormal eddies [24]; (4) selection of a hybrid identification algorithm as the foundational algorithm, followed by multiple validations incorporating the optimization function proposed in this study. The technical workflow of this research is illustrated in Figure 1.

## 2. Study Area

### 2.1. Data

The sources of ocean observation data are extensive, encompassing a wide range of fields and technical methods. Generally, these data are primarily obtained from ocean remote sensing, monitoring conducted by unmanned platforms, and in situ surveys performed by manned vessels. This study necessitates a comprehensive examination of the surface and subsurface characteristics of mesoscale eddies, which requires the collection of a substantial amount of grid data, including multiple layers of water depth, temperature, and salinity. Therefore, this study utilizes the various data types outlined in this section as the foundational raw materials for this research. The data employed in this study spans the period from 1 January 2007 to 31 December 2020 and primarily consists of sea surface height data, thermohaline reanalysis data, and select in situ observation data. This dataset provides a wealth of information regarding the marine environment and significantly enhances the understanding of the dynamic processes of the ocean and their climatic implications.

#### 2.1.1. Ocean Reanalysis Data

The JCOPE2M (Japan Coastal Ocean Predictability Experiment 2 Modified) dataset, meticulously developed by the Japan Marine Affairs Agency, represents a high-resolution reanalysis data product specifically focused on the Northwest Pacific region. This dataset is configured with a temporal resolution of once per day and a spatial mesh accuracy of 1/12°, encompassing 46 levels throughout the entire depth of the ocean. A significant strength of the JCOPE2M dataset lies in its integration of assimilated ocean surface temperature field data, ocean surface altitude anomaly data, and valuable observations from Argo buoys. The amalgamation of these multivariate data sources enhances the dataset’s potential for application and accuracy in various critical areas, including mesoscale eddies, warm salt fields, and flow fields [25]. Numerous studies cited and validated the high accuracy and reliability of the JCOPE2M dataset in related research endeavors.

The Hybrid Coordinate Ocean Model (HYCOM) is a global ocean forecasting model developed by the U.S. Naval Research Laboratory (NRL) that employs an innovative hybrid coordinate system. This model effectively simulates the complex flow patterns present in the upper ocean, thermocline, and even the deep ocean, demonstrating significant applicability at both global and regional scales. HYCOM dataset encompasses a diverse array of predictive elements, including sea surface height, two-dimensional positive pressure flow fields, three-dimensional flow fields, and essential marine environmental parameters such as temperature and salinity. In terms of temporal resolution, the HYCOM dataset achieves a high frequency of hourly updates, while its forecast period is up to seven days, ensuring timely capture and prediction of ocean dynamic changes. In spatial resolution, the dataset maintains 1/12° accuracy on a global scale, and in high-resolution products for specific regions, this accuracy can even reach an amazing 1/25°, greatly improving the precision and applicability of the data. HYCOM also provides a detailed reanalysis data service. With the help of advanced data assimilation technology, the model can combine the observation data of the historical period with the model simulation results to conduct an in-depth reanalysis of the three-dimensional ocean state. This innovative measure not only enriches the database in the Marine field but also provides valuable scientific research materials and sources of inspiration for the majority of scholars [26,27,28].

#### 2.1.2. In Situ Data

The Argo (Array for Real-time Geostrophic Oceanography) floating data set represents a significant international initiative that established the first global array system for the real-time observation of underwater oceanographic data. Since its inception in select regions in 1999, the program has expanded to achieve global coverage within a few years, culminating in the deployment of 3000 buoys by 2007. This development has rendered Argo an essential instrument for investigating alterations in the marine underwater environment [29,30,31].

This study utilized the extensive data resources provided by the Argo real-time data center in China. Specifically, data from 16,351 Argo buoys situated within the KE region was analyzed, covering the period from 1 January 2007 to 31 December 2020. Following a comprehensive quality control process, two primary datasets were identified as follows: 7754 buoys captured by the back air eddy and 5531 buoys captured by the air eddy. To ensure the accuracy and reliability of the data, several key quality control measures were implemented. Firstly, the shallowest data point must be located at a depth greater than 1000 m below the water surface. Secondly, the number of measurement points must not be fewer than 50 within a 1000-m depth to guarantee the adequacy and representativeness of the data. Thirdly, the maximum interval between each measuring point is strictly limited to 20 m to minimize errors associated with uneven spatial distribution. Fourthly, to mitigate the effect of the complex coastal environment, the observation points must be located at least 100 km from the shallow sea edge at a depth of 600 m. These rigorous quality control measures establish a robust foundation for this study, enabling a more precise analysis of ocean dynamic processes and their environmental impacts.

#### 2.1.3. Satellite Observation Data

The Sea Level Anomaly (SLA) and geostrophic current data utilized in this study were sourced from the Archiving, Validation, and Interpretation of Satellite Oceanographic Data (AVISO) provided by the French National Centre for Space Studies. This center is renowned for its high-quality satellite oceanography products, which offer essential data support for global ocean research. SLA and geostrophic flow data from AVISO are meticulously generated by integrating altimetry data from multiple satellites. The raw satellite data undergo a comprehensive processing sequence that includes error correction, data fusion, and spatial interpolation, ultimately resulting in a high-resolution grid of 1/4° × 1/4°. This gridded data format not only enhances data accessibility but also significantly improves its applicability across various oceanographic applications. Regarding the temporal dimension, the original time resolution of AVISO’s SLA and geostrophic data is set at 7 days. However, to accommodate the requirement for high temporal resolution data in this study, interpolation techniques were used to convert these 7-day intervals into a daily frequency. This temporal refinement enables a more precise capture and analysis of the dynamic processes occurring at the ocean surface. In conclusion, SLA and geostrophic current data provided by AVISO, characterized by their high spatial and temporal resolution, as well as superior data quality, establish a robust data foundation for this study, facilitating a deeper exploration of ocean dynamic processes and their associated environmental impacts.

### 2.2. Research Area

**Figure 2:**
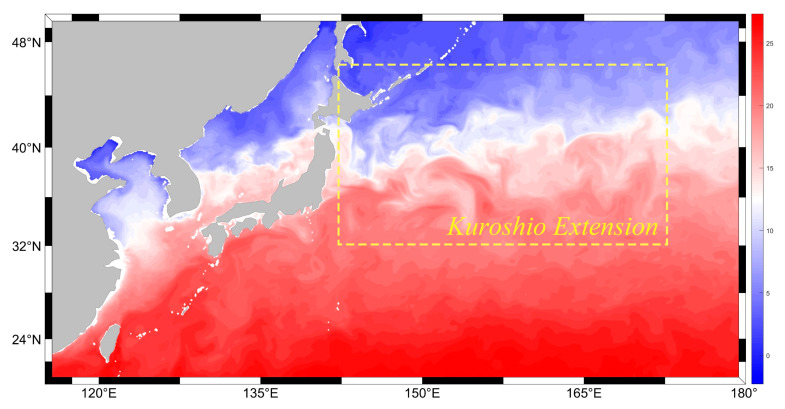
Schematic of the study area.

This study focuses on the three-dimensional reconstruction of mesoscale eddies within the Northwest Pacific Ocean, specifically targeting the KE region (30° N–45° N, 140° E–170° E), an area characterized by a high frequency of mesoscale eddies. All source data described in Section 2.1 are confined to this region. Figure 2 provides a schematic representation of the study area.

## 3. Methods

The objective of this study is to minimize the likelihood of erroneous eddy identification prior to the initiation of the posterior phase of the mesoscale eddy identification process. Consequently, this study does not aim to develop a novel identification algorithm; rather, it acknowledges that the enhanced module requires an existing mesoscale eddy identification algorithm as its foundation. This section utilizes an identification algorithm that is frequently employed by this research group, which has demonstrated notable efficiency and precision in previous applications. This approach, termed a hybrid algorithm, demonstrates superior recognition performance compared to commonly used industry verification methods, as evidenced by preliminary results prior to the completion of this study.

### 3.1. Mesoscale Eddy Hybrid Identification Algorithm

This section introduces a mesoscale eddy hybrid recognition algorithm, serving as the base model for subsequent research. Prior to implementation, the referenced data in Section 2.1 require preprocessing to address the varying resolutions of multi-source inputs. The Akima interpolation method [32] was applied to standardize the data to a grid resolution of 1/8°. Following this, the geostrophic current calculation from the sea surface height data (Equation (1) in Section 2.1.3) was converted into a sea surface flow vector, providing intermediate data for further analysis:

(1) $$u\prime =-\frac{g}{f}\frac{𝜕h\prime }{𝜕y}\;,\;v\prime =-\frac{g}{f}\frac{𝜕h\prime }{𝜕x}$$

where u′ and v′ are the meridional and zonal components of the surface geostrophic flow field (m/s), respectively; and $h^{\prime }$ is the sea surface height (m).

The hybrid algorithm delineates the mesoscale eddy based on the geometric characteristics of the velocity vector, defining it as an approximately circular region where the velocity vector exhibits symmetrical rotation around the eddy center. A region is classified as a potential eddy when the velocity vector field demonstrates rotational flow characteristics. Within this region, the presence of an extreme velocity point, accompanied by a symmetrically distributed velocity vector that rotates either clockwise or counterclockwise around the point, indicates the structure qualifies as a mesoscale eddy. Subsequently, the algorithm employs sea surface height data to identify closed profiles, thereby minimizing the likelihood of identifying non-closed eddies and facilitating the subsequent identification of eddies. Finally, by establishing a custom threshold, the mesoscale eddy information obtained from the preceding two steps is compared systematically. The identification results from both methods are valid only if the intersection area of the two eddies exceeds 50% of the area of a single eddy as determined by each method, and the distance between the eddy centers does not exceed 1/12°. Under these criteria, the eddy center identified through flow field geometry is prioritized as the actual center point.

To assess the efficacy of the hybrid algorithm, this study implemented it alongside the two original algorithms and relevant physical parameters on the same dataset over an equivalent time frame, subsequently submitting the results for expert evaluation. The results are outlined in Table 1. From the perspective of oceanographers, this hybrid algorithm enhances the accuracy and reliability of mesoscale eddy identification, notwithstanding a reduction in the identification rate. This improvement has been substantiated and elucidated in authors’ other studies [33].

### 3.2. Unified Mesoscale Eddy Determination Method

A substantial quantity of identification results will be produced during the identification process of mesoscale eddies. Certain eddies located in the underwater region do not exhibit pronounced characteristics typical of mesoscale eddies; these are referred to as “pseudo-eddies”. This section primarily focuses on the automatic identification of these “pseudo-eddies” by utilizing identification results derived from sea surface data in conjunction with ocean reanalysis data. Additionally, it aims to provide an adequate sample dataset to facilitate the subsequent development of classifiers.

Mesoscale vorticity typically adheres to the significant anomaly rule of thermosalinity within its vertical structure. Cyclones are characterized by a core exhibiting low temperature and low salinity, whereas anticyclonic vorticity is associated with a core that displays high temperature and high salinity. This difference facilitates the automatic extraction and identification of significant mesoscale vorticity features. Therefore, a method was proposed for identifying anomalous thermosalinity profiles of eddy cores. The method begins by extracting the thermohaline anomaly profile at the center of the eddy, utilizing the mesoscale eddy sea surface information obtained in Section 3.1. Subsequently, the climate state signal (specifically, the 30-year monthly average) is removed from this profile to yield the thermohaline anomaly profile at the eddy center, as represented in Equation (2):

(2) $$A=A_{P}-\frac{1}{30}\sum_{i=1}^{30}\sum_{j=1}^{28\sim 31}A_{D}$$

where A is the target thermohaline anomaly profile; $A_{P}$ is the thermohaline profile at the eddy center in the reanalysis data; $A_{D}$ is the daily thermohaline profile of the reanalysis data at the eddy center for 30 years; and i and j are the year and day counts, respectively. Using the statistical results in Zhang et al. [34] regarding the vertical abnormal structure of mesoscale eddies in the KE area, their method was adopted for assessing the significance of the vertical structure of these eddies. This study compared the vertical thermohaline profiles of eddies based on three categories: (1) normal cyclonic (anticyclonic) eddies exhibiting significant underwater structures that correspond with sea surface identification (designated as Normal AE and Normal CE); (2) abnormal AE and abnormal CE eddies, which also display significant underwater structures but do not align with sponge identification results; and (3) eddies classified as significant underwater structures (Table 2). Furthermore, the thermohaline extremum may not coincide with the eddy center at the sea surface due to the vertical axis tilt of the mesoscale eddy. Consequently, relying solely on the thermohaline profile at the eddy center may not adequately represent the vertical anomalies of the eddy. To address this, the research included the average of all profiles within a radius extending outward by 20% from the eddy center in these calculations. Additionally, based on the statistical characteristics of underwater thermohaline anomalies associated with mesoscale eddies, the peak values of core thermohaline anomalies should occur within a specific depth range. This study defines this range using annual statistical results from the Northwest Pacific Ocean (100~500 m). When the vertical thermohaline peaks at the centers of the mesoscale eddies observed within this depth range, they are significant.

**Figure 3:**
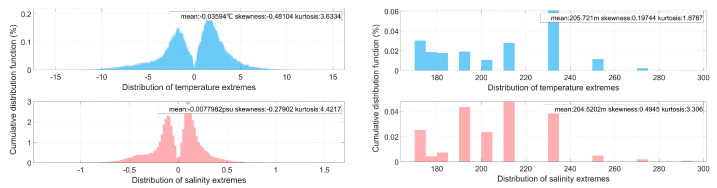
Probability distribution of extreme thermohaline anomaly (**left**) and corresponding depth (**right**) in the KE region.

Figure 3 illustrates the classification of mesoscale vorticity and the statistical results of extreme thermohaline anomalies in the KE region from 1 January 2007 to 31 December 2020 after sea surface identification and underwater structure verification.

Figure 3 depicts the significant thermohaline anomaly values in the KE region, along with the corresponding probability distribution of water depth. The analysis of the thermohaline anomaly distribution indicates that the extreme temperature anomalies associated with gas eddies and anti-gas eddies generally fall within a range of ±5 °C, while the extreme salinity anomalies typically remain within ±0.5 practical salinity units (psu). The probability distribution of these extreme values is concentrated approximately ±2 °C for temperature and ±0.1 psu for salinity. Furthermore, the depth distribution of the thermohaline anomaly extremes reveals that the centers of these anomalies for both types of eddies are predominantly located at approximately 200 m, with a range confined between 150 m and 300 m. In terms of statistical characteristics, the mean values of thermohaline anomalies for gas eddies and anti-gas eddies in the KE region, as selected in this study, exhibit a balance. This balance reflects the closed-loop effect of the properties of the two eddy types on regional phenomena and underscores the conservation of energy transfer within the region.

### 3.3. Portable Mesoscale Eddy Classification Algorithm

Following years of development, the mesoscale eddy identification algorithm has reached a level of maturity. However, existing identification algorithms predominantly rely on the fusion analysis of either single data sets or multi-source data. The separation of these data at the initial level necessitates subsequent re-analysis for integration, which can lead to discrepancies in identification results due to the segmentation between algorithms. This section presents a lightweight and portable identification and classification method, building upon the widely utilized You Only Look Once (YOLO) v11 model. This method enhances the fusion approach for multi-source data collected from the sea surface, thereby facilitating the prior identification of eddies in Section 3.2. The proposed method effectively differentiates between normal and abnormal eddies within mesoscale eddies.

#### 3.3.1. Identification Model

**Figure 4:**
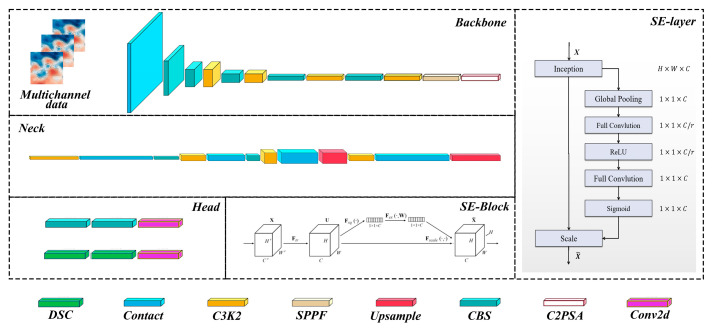
Schematic of the sea surface multi-channel fusion model for mesoscale eddy identification with integrated SE attention mechanism.

Previous methods for the identification of mesoscale eddies predominantly relied on single sea surface height data for analysis. To incorporate additional mesoscale eddy characteristics related to sea surface temperature and salinity, it is essential to utilize multi-source satellite remote sensing data for composite identification. Therefore, the integration of multi-source data is a requisite preparatory step prior to the commencement of the identification process. After thorough deliberation, a multi-source data stack was selected, as illustrated in the multi-channel data input section in Figure 4.

The application of machine vision in the identification of mesoscale eddies has been extensively investigated by numerous scholars over the years. Among the various models explored, the YOLO series has gained significant attention due to its lightweight design and superior cost-effectiveness. Building upon prior research results, this study continues to utilize the YOLO v11 series identification model [35,36]. The latest advancements from Ultralytics have introduced the YOLO v11 model, which achieves enhanced identification performance and improved operational efficiency by modifying the model’s depth and convolutional layers within the backbone, relative to its predecessor, YOLO v8. The YOLO v11 model employs a deep detachable convolutional network as its backbone architecture. A multi-scale extraction approach is implemented within the feature fusion strategy to facilitate the precise identification of small targets. Additionally, a novel multi-task loss feedback mechanism is introduced, which predicts class accuracy and boundary identification errors, thereby enhancing the model’s adaptability. For bounding box losses, YOLO v11 utilizes Complete Intersection over Union (CIoU) and Distribution Focal Loss (DFL) functions, while binary cross-entropy is employed for classification losses. These loss functions significantly improve object identification performance, particularly for smaller objects. In this study, YOLO v11 is selected as the baseline model, comprising three essential components: the backbone network, the neck network, and the predictive output head. The backbone network serves as the core of the YOLO v11 model, tasked with extracting features from input RGB color images. The neck network, positioned between the backbone and the predictive output head, is responsible for aggregating and processing the features derived from the backbone. In YOLO v11, the neck network is pivotal in integrating features across different scales, typically employing a Feature Pyramid Network (FPN) structure that effectively consolidates features at various scales to create a more comprehensive representation. The predictive output head constitutes the uppermost layer of the YOLO v11 model, responsible for identifying and localizing object classes within the image. This output head generally comprises multiple detectors, each designated to predict the position and class of the object. In the YOLO v11 framework, three sets of detectors, each calibrated for different scales, are utilized to assist the model in recognizing objects of varying sizes.

This study aimed to enhance the identification characteristics of mesoscale eddies by improving the foundational model. In addition to incorporating an attention mechanism, the original two-dimensional sea surface height data was expanded to include high-dimensional thermohaline and sea height data. The three-dimensional data was processed using a multi-channel input mode. Furthermore, the SE attention mechanism [37] was integrated to enable the YOLO v11 network to produce different identification results. A schematic representation of the network model is illustrated in Figure 4.

The selection of Squeeze-and-Excitation (SE) attention mechanism is underpinned by two primary reasons. Firstly, SE mechanism enhances the spatial coding quality of images, which can be interpreted as the efficacy of feature map extraction for each channel. Additionally, it facilitates a more explicit adaptive response to the interdependencies among channels. Secondly, this study employs multi-channel data, including sea surface temperature, salinity, and altimetry data, alongside mixed channel inputs. This approach is particularly sensitive to the interdependencies between channels during the identification of mesoscale vorticity, thereby yielding a more effective application for the accurate identification of abnormal mesoscale eddies, as indicated by sea surface data. Figure 4 illustrates the structure of the SE-block within the corresponding module. The input feature graph $X\in R^{H\times W\times C}$ was initially mapped to the feature graph $U\in R^{H\times W\times C}$ by the convolution operation $F_{tr}$. In this process, $V=[v_{1},v_{2},v_{3},\ldots ,v_{C}]$ represents the set of convolution nuclei, and the output can be expressed as $U=[u_{1},u_{2},u_{3},\ldots ,u_{C}]$. Subsequently, the following expression can be presented:

(3) $$u_{c}=v_{c}\;*\;X=\sum_{s=1}^{C}v_{c}^{s}\;*\;x^{s}$$

where * is a convolution; $u_{c}\in R^{H\times W}$; $v_{c}=[v_{c}^{1},v_{c}^{2},\ldots ,v_{c}^{C}]$; $X=[x^{1},x^{2},\ldots x^{C}]$; and $v_{c}^{s}$ is a two-dimensional convolution kernels, with $v_{c}$ acting on a single channel ACTS of X to produce the corresponding channel. To incorporate information from each channel in the output feature map, global spatial information is condensed into a channel descriptor $z_{c}$ using means of global average pooling:

(4) $$z_{c}=F_{sq}\left(u_{c}\right)=\frac{1}{H\times W}\sum_{i=1}^{H}\sum_{j=1}^{W}u_{c}(i,j)$$

To capture channel dependencies, a simple gating mechanism was used with a sigmoid activation function, as follows:

(5) $$s=F_{ex}\left(z,W\right)=\sigma \left(g\left(z,W\right)\right)=\sigma (W_{2}\delta (W_{1}z))$$

where δ is the ReLU function; $W_{1}\in R^{\frac{C}{r}\times C};\;and\;W_{2}\in R^{C\times \frac{C}{r}}$. The final output of the SE block is obtained by rescaling U, as follows:

(6) $${\bar{x}}_{c}=F_{scale}\left(u_{c},s_{c}\right)=s_{c}u_{c}$$

#### 3.3.2. Evaluation Criterion

This study uses the three metrics traditionally utilized by the YOLO series to assess test results: recall rate $\left(R\right)$, accuracy rate $\left(P\right)$, and average accuracy rate $\left(\mathrm{AP}\right)$. The recall rate indicates the proportion of correctly identified targets relative to the total number of actual targets (Equation (7)), while the accuracy rate reflects the proportion of correctly identified targets in relation to the total number of predicted targets (Equation (8)):

(7) $$R=\frac{TP}{TP+FN}\times 100\%$$

(8) $$P=\frac{TP}{TP+FP}\times 100\%$$

where TP is the correctly identified target count; FP is the incorrectly identified target count; FN is the unidentified target count; and average accuracy $\left(\mathrm{AP}\right)$ is a function of accuracy and recall (Equation (9)):

(9) $$AP=\int_{0}^{1}PRdR$$

Given the multiple identification classes in this study, an overall measurement method is required to comprehensively evaluate the identification results across all classes. Evaluation Method mAP@0.5, which has been validated through extensive practical application, is employed for this purpose. The calculation method for Evaluation Method B is presented in Equation (10):

(10) $$mAP=\frac{\sum_{i=1}^{C}AP_{i}}{C}\times 100\%$$

## 4. Experiment

### 4.1. Training

**Figure 5:**
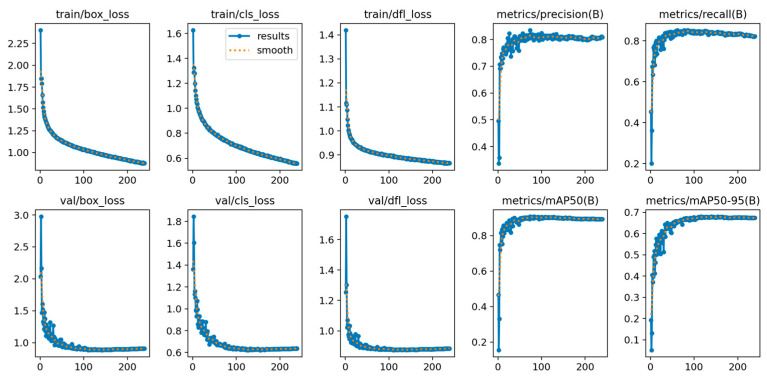
Trends in evaluation metrics during model training and verification.

The classification results obtained in Section 3.2 were utilized to create a sample dataset comprising daily sea surface height data, remote sensing temperature measurements, and sea surface inversion salinity data spanning from 1 January 2007 to 31 December 2020. The time series analysis revealed a total of 5113 data sets, of which 4500 were allocated for training purposes. The remaining 613 data sets were designated as validation data and were employed to train the model described in Section 3.3.1. The trends in accuracy and recall rates, along with the training loss, frame loss, object loss, and class loss, as they relate to the number of epochs, are depicted in Figure 5.

The overall training process of the model exhibits a relatively stable pattern. The training loss demonstrates a slightly oscillatory yet generally stable trend, suggesting that the model’s learning process during training aligns with the typical training dynamics observed in deep learning networks. Furthermore, the results produced by the model hold practical significance.

### 4.2. Evaluation

**Figure 6:**
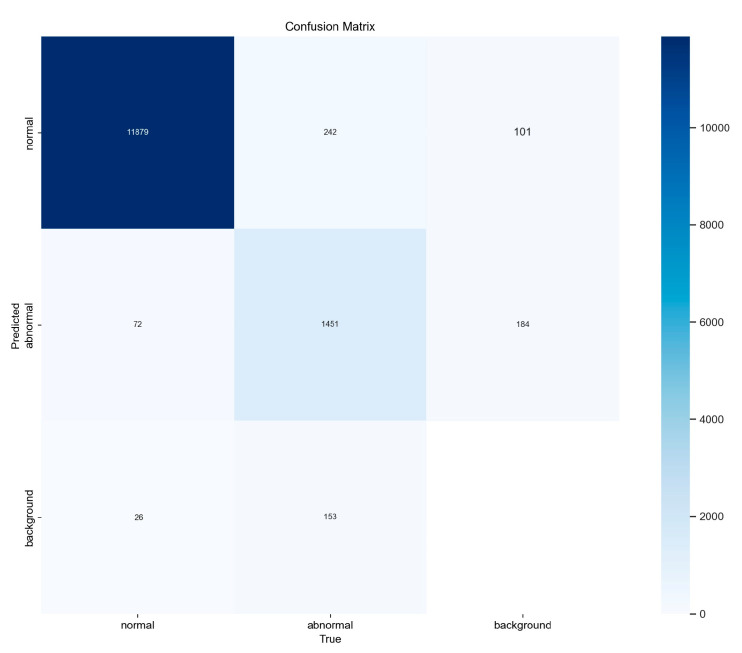
Confusion matrix for model predictions of normal and abnormal eddies against background field.

This section utilizes the multi-source fusion data obtained in Section 3.2 for the purpose of model evaluation. Given that the identification targets exhibit varying quantities in the daily fusion data, the evaluation dataset will be randomly selected and the order of verification will be randomized before being applied to the enhanced model proposed in this study. Figure 6 illustrates the confusion matrix representing the overall identification results of the model. The horizontal axis corresponds to the sample labels designated as actual in the dataset, while the vertical axis reflects the prediction and identification results generated by the model. The grid cells, which exhibit color variations, indicate the identification numbers of mesoscale eddies corresponding to specific longitudinal and latitudinal coordinates. The model demonstrates a higher identification accuracy for normal eddies compared to abnormal eddies, which exhibit slightly inferior identification results. This discrepancy may be attributed to the fact that abnormal eddies do not manifest as prominently as normal eddies in the representation of sea surface features, thereby hindering the model’s ability to effectively learn their characteristics. In Figure 6, the sample size for abnormal eddies is significantly smaller than that for normal eddies. This limited representation of abnormal eddies may result in an insufficient number of samples for effective model fitting, in contrast to the more abundant samples available for normal eddies.

**Figure 7:**
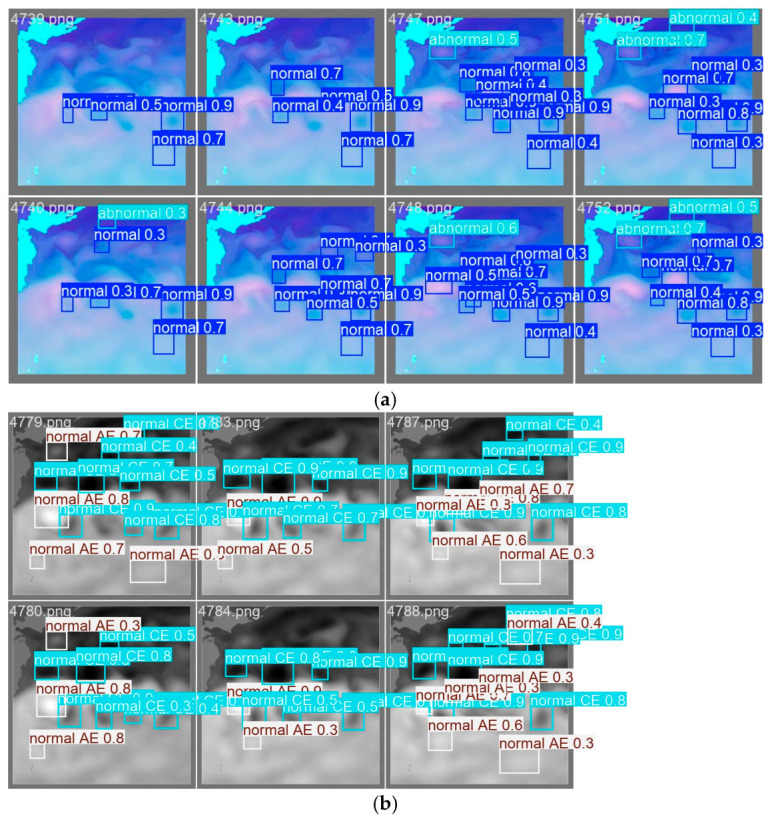
(**a**) Visualization of identification results using 5113 days of daily multi-source remote-sensing superimposed data (2007–2020) based on a sample of 8 consecutive days. (Note: Normal and abnormal eddies are marked without consideration of eddy polarity). (**b**) Visualization of identification results using single sea surface height data.

Figure 7 depicts the identification results of multi-source remote sensing data over a period of eight days. The overall identification performance aligns with expectations. Notably, the identification of eddies adjacent to KE is most successful, whereas the identification of certain eddies within the main body of KE is less effective. In particular, the identification of eddies located within 10 longitudes offshore from KE is the least successful. The regional statistical analyses of the identification results were conducted, revealing that the accuracy of identification within the 140° E–150° E range is only 35%. Furthermore, this study compiled a comparative analysis of the identification and evaluation metrics for this subregion alongside those for the entire study area in tabular format.

It can be clearly seen from Figure 7a that a certain number of abnormal eddies and normal vortices were detected during the 7-day detection period, but the detection of single sea surface height data in Figure 7b failed to identify abnormal eddies.

The results in Table 3 indicate that the prediction accuracy of the model achieves 90% across the entire study area. However, the model’s performance in the offshore (nearshore) region of KE is limited, with an identification accuracy of only 35%. This reduced effectiveness can be attributed to the heightened oceanic activity in this region, which leads to significant fluctuations in the upper sea surface temperature and salinity. Furthermore, following the data fusion process described in this study, the model does not exhibit enhanced eddy characteristics compared to the single-source data, which hinders the identification model’s ability to effectively capture and learn from the data.

### 4.3. Ablation Experiment

This study initially employed fused multi-source remote sensing data as input for the model and subsequently introduced a YOLO eddy identification model enhanced by an integrated attention mechanism. To validate the beneficial effects of these two components during the model training and prediction phases, ablation tests focusing on both aspects were conducted. The fundamental approach involved utilizing the same experimental sample data while systematically controlling the incorporation of the methods. This allowed for a comparative analysis of the identification performance following the enhancements in both areas, thereby demonstrating the efficacy of the improvements made.

This study first examines the effectiveness of multi-source data fusion, utilizing daily sea surface altitude measurement data alongside multi-source fusion data from January 2007 to December 2020 as a control group for the ablation test. The evaluation indicators outlined in Section 3.3.2 were employed as the basis for assessing the results. Furthermore, the effectiveness of the attention mechanism in the SE model is analyzed. To mitigate the potential confounding effects of improved results derived from the fusion data on the ablation test results of this module, experiments using single-source sea surface altimetry data were conducted. The results of these experiments are detailed in Table 4.

In Table 4, the implementation of a multi-source data fusion process, along with the incorporation of a spatial attention mechanism, has significantly enhanced the model’s identification capabilities. Notably, the multi-source data fusion process has markedly improved the identification results for both normal and abnormal eddies. This enhancement can be attributed to the fact that the distinguishing characteristics of normal and abnormal eddies on the sea surface are primarily reflected in the altimetry data, as well as in the temperature and salinity anomalies associated with the eddies. In contrast, relying solely on single altimetry data fails to adequately capture the differences between the two types of eddies, which accounts for the suboptimal identification results in Table 4. Furthermore, while the YOLO model demonstrates certain improvements in identification performance following the integration of the attention mechanism, this enhancement is relatively modest. However, this slight improvement remains of practical significance for applications in eddy identification that necessitate the generation of a substantial volume of identification results.

## 5. Conclusions and Outlook

This study begins by outlining the significance of mesoscale eddies in oceanic processes, drawing upon previous research to establish the background and current state of the field. A review of the development of mesoscale eddy recognition algorithms highlights a key limitation in prior approaches: most traditional algorithms, relying on single-factor data, perform well in identifying eddies on the sea surface but are insufficient for capturing anomalous mesoscale eddy characteristics. To address this, a posterior-guided deep learning network was developed, integrating multi-source data for enhanced identification accuracy. This approach introduces fused data from sea surface altimetry, temperature, and salinity, enabling the retention of multi-feature information on mesoscale eddies through multi-channel input. Identifying the heightened sensitivity between normal and anomalous eddies in these combined data channels (temperature, salinity, sea height), the SE attention mechanism was incorporated into the selected YOLO v11 deep learning model. This integration yielded strong identification results in subsequent verification experiments. Finally, an ablation test demonstrated that the model effectively identifies anomalous eddies while maintaining accuracy in identifying normal eddies, fulfilling the study’s objectives.

Despite these advances, challenges remain, particularly in high-activity regions where intense ocean dynamics complicate identification accuracy for both normal and anomalous eddies. The fusion of multi-source data occasionally introduces inconsistent feature representations, as asynchronous data variations may obscure genuine eddy characteristics. To mitigate this, future work should consider a hybrid approach that integrates the proposed model with established identification algorithms, utilizing the proposed deep learning model to refine and support anomaly identification. (In fact, the mesoscale vortex recognition algorithms mentioned in the introduction can be used as base models). This complementary strategy aims to further optimize identification accuracy, ensuring adaptability across diverse oceanic conditions and advancing the field of mesoscale eddy research.

## Figures and Tables

**Table 1:** Comparative analysis of identification accuracy across mesoscale eddy identification methods.

Methods	Identification Accuracy (%)		Failure to Recognize (%) *	
	AE	CE	AE	CE
Flow field geometry	82.12	76.17	1.52	2.27
Physical parameter	73.24	70.56	2.36	3.52
Closed profile	79.38	79.01	**0.62**	**0.95**
Hybrid (Proposed)	**88.32**	**80.17**	1.97	2.34

Note: * represents eddies that were present but not identified by the respective method.

**Table 2:** Threshold for determining significance of mesoscale eddy underwater structures.

Parameters	Temperature (°C)		Salinity (psu)	
	AE	CE	AE	CE
Underwater anomaly peak	≥+0.3	≤−0.3	≥+0.1	≤−0.1

**Table 3:** Comparison of eddy identification accuracy rates within the complete study area and a selected subregion.

Evaluation Criterion	P	R	MAP@50
Complete area (30° N–45° N, 140° E–170° E)	0.806	0.843	0.902
Partial area (32° N–37° N, 140° E–150° E)	0.415	0.494	0.346

**Table 4:** Ablation test results comparing multi-source fusion data and SE attention mechanism effect.

Evaluation Criterion	P	R	MAP@50
Single data with YOLO v11 (SSH)	0.806	0.843	0.902
Multi data with YOLO v11 (Temperature, Salinity, SSH)	**0.415**	**0.494**	**0.346**
YOLO v11	0.752	0.801	0.874
YOLO v11 with SE attention	**0.806**	**0.843**	**0.902**

## Data Availability

The data used in this paper are available from publicly available institutions and are cited in this paper.
